# ﻿A new species of beaded lacewings (Neuroptera, Berothidae) from mid-Cretaceous Myanmar amber

**DOI:** 10.3897/zookeys.1092.79396

**Published:** 2022-04-04

**Authors:** Sihang Zhang, Yihong Yang, Jiayi Chen, Liming Liu, Zhendong Cao, Sanping Xie

**Affiliations:** 1 School of Earth Sciences and Key Laboratory of Mineral Resources in Western China (Gansu Province), Lanzhou University, Lanzhou 730000, China Lanzhou University Lanzhou China

**Keywords:** fossil, lower Cenomanian, Mantispoidea, Neuropterida, taxonomy

## Abstract

A new species of Berothidae, *Jersiberothamusivum* sp. nov., is described and illustrated from mid-Cretaceous (lowest Cenomanian) Myanmar amber. It is easily distinguished from other species of Berothidae by the configuration of the wing venation including: forewing with distinct areas of infuscation surrounding cross-veins and vein forks, all cross-veins simple prior to ScP-RA fusion, presence of two cross-veins ra-rp; absence of inner or outer graduate series of cross-veins; RP with three branches; and absence of ma-mp cross-veins and cua-cup cross-veins; while hind wing has cross-vein 1r-m absent. The previous diagnoses of *Iceloberotha* Grimaldi, 2000 and *Jersiberotha* Grimaldi, 2000 are quite unclear because some characters occur mosaically in both genera. In order to solve this problem and distinguish *J.musivum* from other species in the family, a new key to species of Berothidae from Myanmar amber has been provided and the diagnoses of *Iceloberotha* and *Jersiberotha* have been revised.

## ﻿Introduction

The family Berothidae, commonly known as beaded lacewings, together with Mantispidae and Rhachiberothidae, belongs to the superfamily Mantispoidea, which represents one of the major lineages of the crown group of Neuroptera (Wang et al. 2016; Engel et al. 2018; Winterton et al. 2018). The extant Berothidae are distributed in all zoogeographical regions and currently comprise less than 130 described species of 28 genera worldwide (Oswald 2019). The genus *Jersiberotha* was firstly erected by Grimaldi from the New Jersey amber for sharing the wing characters: two adjacent ra-rp cross-veins in the forewing, only one ma- mp cross-vein (Grimaldi 2000). Engel and Grimaldi (2008) described two other species (*J.myanmarensis* and *J.tauberorum*) and a related genus *Iceloberotha* from Myanmar amber.

Molecular evidence suggested the Berothidae might have diverged during the Late Triassic (Wang et al. 2016). Winterton et al. (2018) and Vasilikopoulos et al. (2020) estimated the divergence between Beorthidae and Mantispidae was in the Early Jurassic, although the oldest definite fossil of Berothidae was reported from the Middle Jurassic (Makarkin et al. 2011). So far, there are about 27 genera and 41 species of fossil berothids described from the Middle Jurassic to late Eocene (Yang et al. 2019; Fuente et al. 2021). The amber from Myanmar is one of the most intensively studied objects amongst Cretaceous fossiliferous resins and contains a remarkably diverse flora and fauna (Grimaldi et al. 2002). It is remarkable that the palaeodiversity of Berothidae is extraordinarily rich from the mid-Cretaceous of Myanmar, being one of the major Neuropteran lineages in this deposit (Ross 2019), with 13 genera and 19 species described, based on the Myanmar amber specimens (Engel and Grimaldi 2008; Yuan et al. 2016; Huang et al. 2018; Makarkin 2018; Yang et al. 2019; Yang et al. 2020). These Myanmar amber berothids show diverse morphological characters and are probably the members, or as the stem-groups of different subfamilies (Huang et al. 2018).

Herein, a new species of *Jersiberotha* Grimaldi, 2000 is described, based on a well-preserved female specimen from the mid-Cretaceous (ca. 98.8 Ma) of Myanmar. In addition, a revised key to the species of Berothidae from Myanmar amber has been provided. The diagnoses of *Iceloberotha* and *Jersiberotha* also have been revised.

## ﻿Materials and methods

This study is based on a single, female specimen preserved in a piece of clear, yellowish Myanmar amber, which was collected from an amber mine, located in the Hukawng Valley of Kachin Province, Myanmar (Chen et al. 2019). The age of Myanmar amber in this investigation is radiometrically dated at 98.79 ± 0.62 Ma, based on U-Pb zircon dating of the volcanoclastic matrix (Shi et al. 2012). However, it is important to highlight that the Zircon date only gives the age of the top bed and a minimum age for the amber, so the geologic age of Myanmar amber may be slightly older than the Zircon date.

The amber piece that contains the inclusions was cut and polished with different grain sizes of sandpaper and finally buffed with polishing powder. The specimen is housed in the Institute of Paleontology and Stratigraphy, School of Earth Sciences, Lanzhou University, Gansu Province, China. Examinations of the specimen were accomplished using a Leica S8APO stereomicroscope, equipped with a Leica DFC295 camera. Images were digitally stacked as photomicrographic composites of approximately 20 individual focal planes obtained using the software Helicon Focus 6 for better visualisation of the 3D structures. Drawings for the analysis were based on the specimen and photographs. Figures were prepared with CorelDraw X4 and Adobe Photoshop CS6.

General terminology of wing venation follows Kukalová-Peck and Lawrence (2004) as interpreted by Yang et al. (2012) and Yang et al. (2014). Terminology of the wing spaces and details of venation (e.g. spaces, veinlets) follows Oswald (1993) and the cross-veins designation follows Makarkin (2015). Terminology of genitalia follows Aspöck and Aspöck (2008). Cross-veins are designated after the longitudinal veins which they connect and are numbered in sequence from the base to the apex of the wing, for example, 1sc-r, first (proximal-most) cross-vein connecting Sc and R; 2m-icu, second cross-vein between M/MP and Cu/CuA.

Abbreviations are as follows: wing venation: ScA, subcosta anterior, ScP, subcosta posterior, RA, anterior radius, RP, posterior sector, RP1, proximal-most branch, MA and MP, anterior and posterior branches of media, CuA, anterior cubitus, CuP, posterior cubitus, AA1-AA3, first to third anterior anal vein; head and antennal structures: La, labrum, MP, maxillary palp, Pe, pedicel, Sp, scape; abdominal and genital structures: T: Tergite; S: sternite; phc: pseudohypocaudae; e: ectoproct; gcx, gonocoxites.

The identification key to the Berothidae species from Myanmar amber was modified from that provided by Yuan et al. (2016), based on the characters of the new species.

## ﻿Systematic palaeontology

### ﻿Order Neuroptera Linnaeus, 1758

**Family Berothidae Handlirsch, 1906**

#### Genus *Jersiberotha* Grimaldi, 2000

##### ﻿ Jersiberotha musivum sp. nov.

Taxon classificationAnimaliaNeuropteraBerothidae

05CA0583-678B-50A2-88C7-B96EB206D15D

http://zoobank.org/76B6E201-6262-4FC0-ACAC-1FF50C04815C

###### Diagnosis.

*Jersiberothamusivum* may be easily distinguished from the other four species of *Jersiberotha* by a combination of the following character states: in forewing, two cross-veins ra-rp are present; inner or outer graduate series of cross-veins are absent; RP has three branches; ma-mp cross-veins and cua-cup cross-veins are absent; and basal cross-vein 1r-m is absent from hind wing.

###### Etymology.

The specific name is from the Latin “*musivum*” (meaning “mosaic”), in reference of the new species with some characters in wing venation occurring mosaically in both two similar genera, i.e. *Iceloberotha* and *Jersiberotha*.

###### Locality and horizon.

Noije Bum Hill, Hukawng Valley, Kachin State, Myanmar; lower Cenomanian, mid-Cretaceous.

###### Material examined.

***Holotype*** LZUGSW20210219; deposited in the Institute of Paleontology and Stratigraphy, School of Earth Sciences, Lanzhou University, Gansu Province, China.

###### Description.

**Figure 1.**
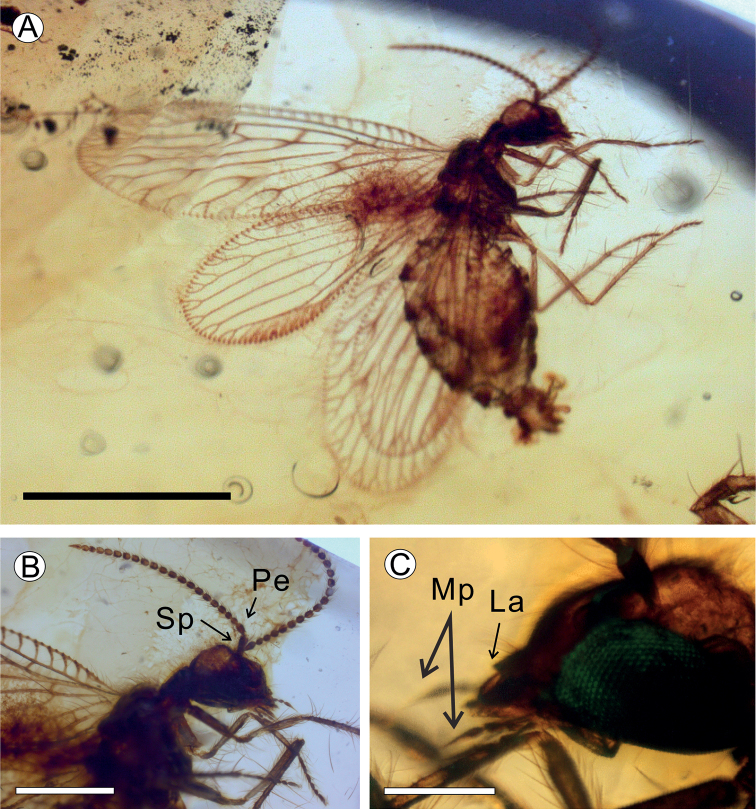
*Jersiberothamusivum* sp. nov. Holotype LZUGSW20210219 **A** photograph of holotype habitus (lateral left view) **B** photograph of head, pedicel (Pe) and scape (Sp) **C** photograph of antenna, maxillary palpus (Mp) and labrum (La). Scale bars: 1 mm (**A**); 200 μm (**B**); 100 μm (**C**).

**Female** (Fig. 1A). Body length ca. 1.50 mm as preserved. ***Head***: oval in lateral view, length ca. 0.2 mm. Compound eyes prominent, ovoid, large, length ca. 0.14 mm. Vertex with sparse long setae. 3^rd^ and 4^th^ segments of maxillary palpus relatively short, approximately twice as long as wide; apical tarsomere elongated, very acute distally, approximately five times as long as wide. Labial palpus not fully visible. Antenna: length ca. 0.5 mm (Fig. 1B), scapus elongate, approximately three times as long as maximum width; pedicellus elongate, approximately two times longer than maximum width and slightly broader than flagellomeres; the following 21 flagellomeres, cylindrical, slightly longer than wide, with relatively long setae around; basal flagellomeres transverse, distal flagellomeres slightly elongate; apical flagellomere conical.

**Figure 2.**
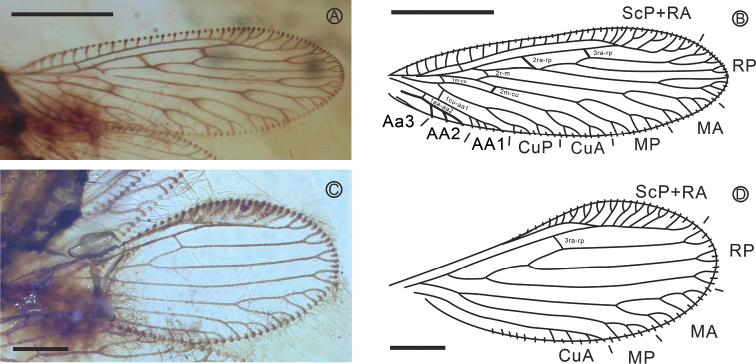
Wing venation of *Jersiberothamusivum* sp. nov., Holotype LZUGSW20210219 **A** photograph of forewing **B** line drawing of forewing **C** photograph of hind wing **D** line drawing of hind wing. Scale bars: 500 μm (**A, B**); 200 μm (**C, D**).

***Forewing***: narrowly slender and oval shaped (Fig. 2A, B), length ca. 1.70 mm, width ca. 0.55 mm. Leading edge with short setae, trailing edge with long setae. Costal space narrow, slightly narrowed medially. All subcostal veinlets simple, basal and distal subcostal veinlets slightly curved. Humeral veinlet cross-vein-like. Subcostal space nearly as broad as costal space, proximal cross-vein (1scp-r) located proximad at origin of RP; ScP terminating at RA. ScP+RA entering margin before wing apex, shallowly forked distally with five short branches. RA space a little broader than subcostal space, with only two cross-veins present, the basal one proximad to middle of wing and the distal one proximad at fusion of ScP and RA. RA slightly thickened; RP originating from RA relatively distant from wing base, with two long branches originating between 2ra-rp and 3ra-rp and one short branch distad at 3ra-rp. RP1 with 2 branches, primary fork distad at 3ra-rp, one branch not forked, other with three pectinate distal branches; stem of RP, RP2 and RP3 shallowly forked. No cross-veins between branches of RP. M fused with R basally. One cross-vein between RP and M (2r-m) connecting stem of RP and MA. M basally approaching R; forked nearly at origin of RP. MA forked primarily distad at 3ra-rp, proximal branch with two pectinate distal branches, distal branch once forked distally; MP forked primarily between 2ra-rp and 3 ra-rp, proximal branch with two pectinate distal branches, distal branch once forked distally. No cross-veins between MA and MP. Two cross-veins between M and Cu: 1m-cu connecting M and Cu distad at origin of CuP; 2m-cu connecting MP and CuA. Cu divided into CuA and CuP rather far from wing base. CuA and CuP dichotomously branched. No intracubital cross-veins. One cross-vein (1cu-aa1) between Cu and AA1 connecting CuP, AA1 much proximad to their branching. AA1 once forked distally, AA2 thickened, pectinately branched, with two short branches. AA3 short, simple. Marginal setae long to very long and arranged in bunches at end of veins and trichosors. Trichosors prominent along entire wing margin. Wing membrane hyaline, with following dark brown maculation; narrowly margined cross-vein 2ra-rp, 3ra-rp and 2r-m; widely margined cross-vein 2m-cu; spots at origin (inside) of RP, RP1, RP3 and at primary forks of RP1, MA, MP, CuA and CuP.

***Hind wing***: narrowed proximally, broadened distally, length ca. 1.40 mm, width ca. 0.55 mm (Fig. 2C, D). Costal space strongly narrowed medially, slightly dilated proximally and strongly dilated distad at fusion of ScP and RA. Subcostal veinlets simple, closely spaced; distal subcostal veinlets strongly oblique, thickened. Subcostal space rather broad, basally appears narrowed; no cross-veins detected. ScP stout, nearly straight before terminating at RA, ScP + RA entering margin far before wing apex, veinlets are difficult to be accurately counted, but it appears there are six veinlets. RA space clearly wider than subcostal space, with one cross-vein proximad to fusion of ScP and RA, none distad. RP originating rather far from wing base, with two branches originated proximad to 3ra-rp, none distad. Stem of RP, RP1 and RP2 shallowly once forked. No cross-veins between branches of RA, RP and M; basal 1r-m absent. M basally fused with R; forked distad origin of RP. MA and MP nearly parallel for most of the length; MA dichotomously branched; MP pectinately branched, with two short simple branches, no intramedian cross-veins. Cross-veins between M, Cu (1m-cu) not discernible. CuA pectinately branched, with three short simple branches. CuP and AA1 not detected. Marginal setae very long, arranged in bunches at end of veins, especially along hind margin. Wing membrane hyaline, without maculation.

**Figure 3.**
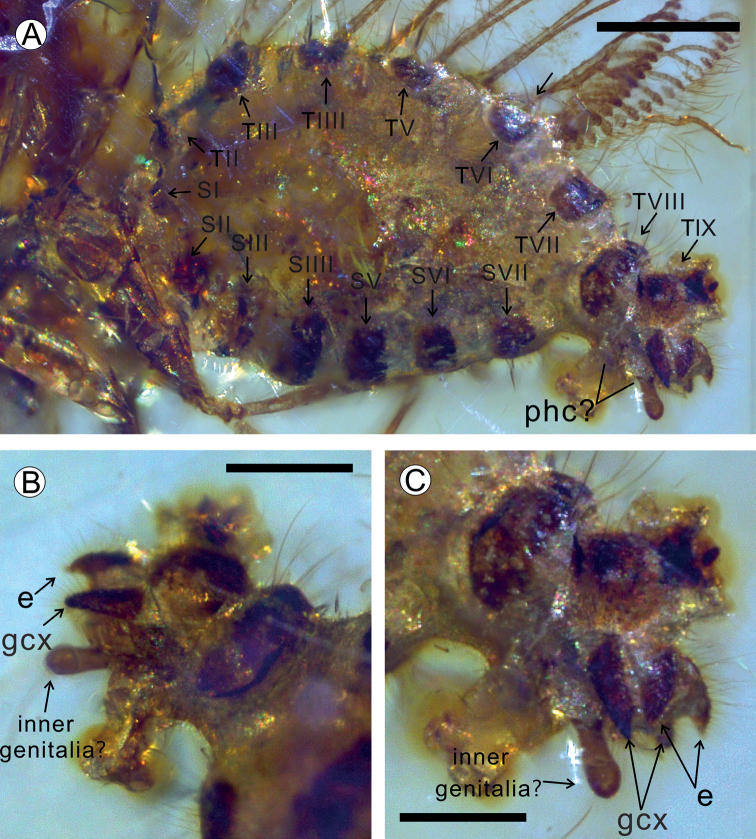
Abdomen and genital of *Jersiberothamusivum* sp. nov., Holotype LZUGSW20210219 **A** abdomen, lateral view **B** terminal segments, ventro-lateral view **C** terminal segments, lateral view. Scale bars: 200 μm (**A**); 100 μm (**B, C**).

***Abdomen*** oval, stout. All segments clearly visible except terminal-most, with broad membranous space between sternites and tergites (Fig. 3A). Tergite I not visible, Tergite II and sternite II shorter than tergites and sternites of abdominal segments III – VII, without specialised modifications. Tergite VIII well developed, dorsally broader than laterally, elongate and extending towards the sternite. Tergite IX not clearly identified, sternite VIII reduced. The genitalia structures of the new species are not well preserved, the pair of ectoprocts (e) are slightly shorter than the other pair of gonocoxites (gcx) and the unpaired club-shape process may be a part of inner genitalia (Fig. 3B, C). All tergites and sternites covered with dense, long fine setae.

## ﻿Discussion

### ﻿Key to species of Berothidae from Myanmar amber

**Table:** 

1	Cross-veins ra-rp present in forewing; forewing typically with parse setae, rarely with dense setae	**2**
–	Cross-veins ra-rp absent in forewing; forewing very densely covered with elongate setae	***Ethiroberothaelongata* Engel & Grimaldi, 2008**
2	Only one ra-rp cross-vein	**3**
–	More than one ra-rp cross-vein (at least two)	**5**
3	Flagellum with 21-22 flagellomeres	**4**
–	Flagellum with more than 70 flagellomeres	***Maculaberotha* , Yuan, 2016**
4	Vein RP with only one branch in forewing	***Haploberothapersephone* Engel & Grimaldi, 2008**
–	Vein RP with three branches in forewing	***Haploberothacarsteni* Makarkin, 2018**
5	Distalmost ra-rp cross-vein present beyond Sc-R1 fusion in forewing	**6**
–	Distalmost ra-rp cross-vein present before Sc-R1 fusion in forewing	**7**
6	Forewing with numerous setae on margins, sparse over wing surface; not obscuring wing venation; apical rp-rp and rp-m cross-veins absent. Body setae short and scattered	***Telistoberothalibitina* Engel & Grimaldi, 2008**
–	Forewing with dense setae on margins and over surface; apical rp-rp and rp-m present. Body setae dense and elongate	***Dasyberothaeucharis* Engel & Grimaldi, 2008**
7	Forewing narrowly elongate, apex acute; costal space considerably broader basally than apically at ScP-RA fusion; scape short, as long as wide	***Systenoberothamagillae* Engel & Grimaldi, 2008**
–	Forewing ovoid, apex broadly round; costal space not broader basally than apically at ScP-RA fusion; scape 2-3 times as long as wide	**8**
8	Humeral vein recurrent	***Magniberotharecurrens* Yuan, 2016**
–	Humeral vein simple, not recurrent	**9**
9	Flagellum with more than 70 flagellomeres	**10**
–	Flagellum with 21-22 flagellomeres	**15**
10	Forewing with 4-5 ra-rp cross-veins proximad to fusing point of ScP and RA	.**11**
–	Forewing with 2-3 ra-rp cross-veins proximad to fusing point of ScP and RA	**14**
11	Frons without a pair of short horns anteriad to antennal bases	***Ansoberothajiewenae* Yang, 2019**
–	Frons with a pair of short horns anteriad to antennal bases	**12**
12	Vein RP with eight branches	***Cornoberothamonogona* Yang, 2020**
–	Vein RP with less than eight branches	**13**
13	Vein RP with five branches	***Cornoberothaaspoeckae* Yang, 202**0
–	Vein RP with six branches	***Cornoberothaanomala* Yang, 2020**
14	Vein RP with six branches	***Dolichoberothabifurcate* Yang, 2020**
–	Vein RP with four branches	***Dolichoberothaburmana* Yang, 2020**
15	Forewing with distinct areas of infuscation surrounding cross-veins and vein forks, some or all cross-veins simple prior to ScP-RAfusion	**16**
–	Forewing lacking areas of infuscation; all c-sc cross-veins simple prior to ScP-RAfusion	**18**
16	Forewing with one ma-mp cross-vein and one cua-cup cross-vein present	***Jersiberothamyanmarensis* Engel & Grimaldi, 2008**
–	Forewing with ma-mp cross-vein and cua-cup cross-vein absent	**17**
17	Hind wing with basal cross-vein 1r-m present	***Jersiberothatauberorum* Engel & Grimaldi, 2008**
–	Hind wing with basal cross-vein 1r-m absent	***Jersiberothamusivum* sp. nov.**
18	Forewing with outer gradate series of cross-veins present	***Iceloberothakachinensis* Engel & Grimaldi, 2008**
–	Forewing with outer gradate series of cross-veins absent	***Iceloberothasimulatrix* Engel & Grimaldi, 2008**

The new species *Jersiberothamusivum* sp. nov. can be assigned to the family Berothidae, based on the forked veins in subcostal region and the scape three times as long as wide. A new identification key to the species of Berothidae from Myanmar amber has been presented and compared with the new species of other ones of this family. The venation of *Jersiberothamusivum* sp. nov. differs from that of *Protoberotha* Huang, 2018 and *Ethiroberotha* Engel & Grimaldi, 2008 in presence of cross-veins ra-rp in forewing, which is absent in both above-mentioned genera. The new species has more than one ra-rp cross-veins in forewing, differentiating it from the representatives of *Haploberotha* Engel & Grimaldi, 2008, which only has one ra-rp cross-vein.

The species of *Maculaberotha* Yuan, 2016 has RP with four branches in forewing and more than 70 flagellomeres, while the *J.musivum* sp. nov. possesses RP with three branches and flagellum with 21 flagellomeres. The distalmost ra-rp cross-vein before ScP-RA fusion in forewing differs that of *Telistoberotha* Engel & Grimaldi, 2008 and *Dasyberotha* Engel & Grimaldi, 2008, which have the cross-vein ra-rp beyond ScP-RA fusion.

*Jersiberothamusivum* sp. nov. also differs from that of *Systenoberotha* Engel & Grimaldi, 2008 in the scape three times as long as wide, in contrast to the scape being as long as wide in *Systenoberotha*, from *Magniberotha* Yuan, 2016 in humeral vein simple, not recurrent and RP with three branches in forewing, in contrast to the humeral vein being recurrent and RP with five branches in *Magniberotha*, as well as from that of *Ansoberotha* Yang, 2019, *Cornoberotha* Yang, 2020 and *Dolichoberotha* Yang, 2020 in flagellum with 21 flagellomeres, in contrast to the flagellum having more than 70 flagellomeres in all the three above-mentioned genera.

Amongst all known members of Berothidae, the venation of *Jersiberothamusivum* sp. nov. is more similar to that of *Iceloberotha* Engel & Grimaldi, 2008. *Jersiberotha* and *Iceloberotha* were initially distinguished, based on the obviously diagnostic characters of venation, i.e. speckless forewing in *Iceloberotha* vs. distinctly spotted forewing in *Jersiberotha*; absence of cross-vein ma-mp and cua-cup in *Iceloberotha* vs. presence of ma-mp and cua-cup in *Jersiberotha* (referring to the key to genera in Engel and Grimaldi 2008). Actually, these characters occurred mosaically in both genera, for example, the Myanmar species *J.tauberorum* Engel & Grimaldi, 2008 clearly loses the cross-veins ma-mp and cua-cup, while the type species of *Iceloberotha* has an evident ma-mp cross-vein in the original drawings (Engel and Grimaldi 2008). The new species can be assigned to *Jersiberotha*, based on flagellum with 21 flagellomeres, forewing with distinct areas of infuscation surrounding cross-veins and vein forks, all cross-veins simple prior to ScP-RA fusion, presence of two cross-veins ra-rp, absence of inner or outer graduate series of cross-veins; RP with three branches.

The venation of the new species clearly differs from that of *Iceloberotha* Engel & Grimaldi, 2008 in forewing with distinct areas of infuscation surrounding cross-veins and vein forks, but the representatives of *Iceloberotha* lack areas of infuscation and all c-sc cross-veins are simple prior to ScP-RA fusion. *J.musivum* sp. nov. also differs from that of *J.myanmarensis* Engel & Grimaldi, 2008 and two New Jersey species (*J.luzzii* Grimaldi, 2000 and *J.similis* Grimaldi, 2000) in forewing with ma-mp cross-vein and cua-cup cross-vein absent, with the forewing with ma-mp cross-vein and cua-cup cross-vein being present in all three above-mentioned genera, as well as from *J.tauberorum* that has a 1r-m cross-vein on hind wing and cross-vein absent in the new species. Additionally, the absence of outer gradate series of cross-veins in the new species is similar to that of *I.simulatrix* Engel & Grimaldi, 2008 and *J.tauberorum* Engel & Grimaldi, 2008. The loss of the gradate series is not found in extant berothids (Makarkin 2018).

In summary, the above analysis indicates the fossil specimen here is most closely related to the genus *Jersiberotha* due to the observed combination of character states. The previous diagnoses of *Jersiberotha* and *Iceloberotha* are quite unclear because some characters occur mosaically in both genera. The diagnosis of *Jersiberotha* should be revised as flagellum with 21–22 flagellomeres, forewing with distinct areas of infuscation surrounding cross-veins and vein forks, some or all cross-veins simple prior to ScP-RA fusion, presence of more than one cross-veins ra-rp, absence of inner or outer graduate series of cross-veins; RP with no more than three branches and the diagnosis of *Iceloberotha* should be revised as flagellum with 21–22 flagellomeres, forewing without distinct areas of infuscation surrounding cross-veins and vein forks, all cross-veins simple prior to ScP-RA fusion, presence of more than one cross-veins ra-rp, RP with no more than three branches.

## ﻿Conclusions

*Jersiberothamusivum* sp. nov. was described, based on a single, female from mid-Cretaceous Myanmar amber. The previous diagnosis between *Iceloberotha* and *Jersiberotha* was quite indistinct because some characters occurred mosaically in both genera. A new identification key to the Berothidae species from Myanmar amber was provided to distinguish the new species from others in the Berothidae and the revised diagnoses of *Iceloberotha* and *Jersiberotha* were also updated.

## Supplementary Material

XML Treatment for Jersiberotha musivum
